# Treatment of Neuralgia, by the Valerianate of Ammonia

**Published:** 1857-01

**Authors:** 


### *Treatment of Neuralgia, by the Valerianate of Ammonia.*
By Dr. Declat.

We have prepared an abstract of an interesting translation from the Revue Med. and Etrangue, which may be found in a late number of the Medical Examiner, as it brings to our notice a new remedy, which may be of value in the treatment of a class of diseases increasing in frequency and oftentimes obstinate in their persistency. According to Dr. Declat, such cases will yield to the influence of valerianate of ammonia; and as proof of his statement, he gives the following cases:

Case I. The marchioness of Fontanelle suffered with facial neuralgia for six years, first appearing as she was cutting a wisdom tooth. Legrand and Jobert (de Lamballe) ordered its extraction, which was done, causing agonizing pain. The neuralgia still continued in spite of every effort of such advisers as Sedillot, Velpeau and Jobert. Quinine, opium, belladonna, strychnia, iron, gold and quinquina were employed, and external applications, such as blisters, opium plasters, dulcamara, chloroform, collodin, aconite, &c. Everything failed. Jobert applied the actual cautery along the course of the inferior maxillary nerve, and after trying the waters of Plombiere with partial though temporary relief, the Marchioness applies to Dr. Declat.

The first remedy used was Fowler's solution, which was pushed until it produced constitutional symptoms, without success. The patient had become almost insane from the agony, when on experiment was made with valerianate of ammonia, on the 3d of January. A teaspoonful that night relieved partially, and two teaspoonfuls the next day entirely banished the pain. The medicine was discontinued May 6th. Occasionally, however, Mad. Ferrand has "slight twinges," but resorts to the specific, and always successfully. This lady seems to have hereditary right to neuralgia, her mother having been a great victim to the disease, whilst her brother, the Earl of Essex, has had tic doloureux from his youth.

Case II. M. Letellier, who had suffered horribly with pain in the head, extending to the neck, and losing itself on the branches of the facial nerve, was at Plombiere's when taken, and returned to Paris in great agony. Dr. Louis tried blisters, sage, quinine and morphia without any effect. He used morphia to such excess as to remain in stupor almost constantly. Dr. Declat administered the valerianate of ammonia in drachm doses, twice a day. In five days he was up, and in nine days all pain had passed away. He has since stated that his cure was complete.

